# Multi-omics analysis to examine microbiota, host gene expression and metabolites in the intestine of black tiger shrimp (*Penaeus monodon*) with different growth performance

**DOI:** 10.7717/peerj.9646

**Published:** 2020-08-14

**Authors:** Tanaporn Uengwetwanit, Umaporn Uawisetwathana, Sopacha Arayamethakorn, Juthatip Khudet, Sage Chaiyapechara, Nitsara Karoonuthaisiri, Wanilada Rungrassamee

**Affiliations:** 1 Microarray Research Team, National Center for Genetic Engineering and Biotechnology, Pathum Thani, Thailand; 2 Shrimp Genetic Improvement Center, National Center for Genetic Engineering and Biotechnology, Pathum Thani, Thailand; 3 Aquaculture Service Development Research Team, National Center for Genetic Engineering and Biotechnology, Pathum Thani, Thailand

**Keywords:** Microbiota, Transcriptome, Metabolites, Shrimp intestine, Black tiger shrimp, Growth performance, *Penaeus monodon*

## Abstract

Understanding the correlation between shrimp growth and their intestinal bacteria would be necessary to optimize animal’s growth performance. Here, we compared the bacterial profiles along with the shrimp’s gene expression responses and metabolites in the intestines between the Top and the Bottom weight groups. Black tiger shrimp (*Penaeus monodon*) were collected from the same population and rearing environments. The two weight groups, the Top-weight group with an average weight of 36.82 ± 0.41 g and the Bottom-weight group with an average weight of 17.80 ± 11.81 g, were selected. Intestines were aseptically collected and subjected to microbiota, transcriptomic and metabolomic profile analyses. The weighted-principal coordinates analysis (PCoA) based on UniFrac distances showed similar bacterial profiles between the two groups, suggesting similar relative composition of the overall bacterial community structures. This observed similarity was likely due to the fact that shrimp were from the same genetic background and reared under the same habitat and diets. On the other hand, the unweighted-distance matrix revealed that the bacterial profiles associated in intestines of the Top-weight group were clustered distinctly from those of the Bottom-weight shrimp, suggesting that some unique non-dominant bacterial genera were found associated with either group. The key bacterial members associated to the Top-weight shrimp were mostly from Firmicutes (*Brevibacillus* and *Fusibacter*) and Bacteroidetes (*Spongiimonas*), both of which were found in significantly higher abundance than those of the Bottom-weight shrimp. Transcriptomic profile of shrimp intestines found significant upregulation of genes mostly involved in nutrient metabolisms and energy storage in the Top-weight shrimp. In addition to significantly expressed metabolic-related genes, the Bottom-weight shrimp also showed significant upregulation of stress and immune-related genes, suggesting that these pathways might contribute to different degrees of shrimp growth performance. A non-targeted metabolome analysis from shrimp intestines revealed different metabolic responsive patterns, in which the Top-weight shrimp contained significantly higher levels of short chain fatty acids, lipids and organic compounds than the Bottom-weight shrimp. The identified metabolites included those that were known to be produced by intestinal bacteria such as butyric acid, 4-indolecarbaldehyde and L-3-phenyllactic acid as well as those produced by shrimp such as acyl-carnitines and lysophosphatidylcholine. The functions of these metabolites were related to nutrient absorption and metabolisms. Our findings provide the first report utilizing multi-omics integration approach to investigate microbiota, metabolic and transcriptomics profiles of the host shrimp and their potential roles and relationship to shrimp growth performance.

## Introduction

Fisheries and aquaculture production play pivotal role to provide food sources for increasing global population. In 2016, fisheries have contributed 90.9 million tons and aquaculture supplied 80.0 million tons of food supplies for human consumption (FAO, 2018a). Crustaceans are accounted for 7.9 million tons, in which approximately 70% of crustaceans are from shrimp and prawn production (FAO, 2018b). Black tiger shrimp (*Penaeus monodon*) is one of the main species for shrimp aquaculture in South East Asia, the coasts of Australia and East Africa. However, there are several challenges such as breeding difficulties and disease outbreaks which, if not addressed, could impede the sustainable development of shrimp aquaculture and food security (Marsden et al., 2013; Stentiford et al., 2012).

To improve shrimp production, an alternative approach to improve shrimp growth performance is crucial. Microbiome plays essential roles in host health such as nutrient acquisition, physiological adaptation and immune system (Hanning & Diaz-Sanchez, 2015). Therefore, applications of beneficial bacteria and modulation of intestinal microbiota to improve health and immune responses have been promising means to enhance growth performance and disease resistance in shellfish aquaculture (Ringo , 2020; Wang et al., 2020). For instance, *Lactobacillus* species as feed additives has been reported to improve immune responses and modulate gut microbiota in marron (*Cherax cainii*) (Foysal et al., 2020), improve immune response and disease resistance in Pacific white shrimp (*Litopenaeus vannamei*) (Roomiani, Ahmadi & Ghaeni, 2018), increase growth performance in giant river prawn (*Macrobrachium rosenbergii*) (Karthik & Bhavan, 2018), and enhance immune-related enzymes in crayfish (*Astacus leptodactylus*) (Valipour et al., 2019). Additionally, shrimp feed supplemented with *Bacillus* has been shown to improve survival under pathogen exposure in black tiger shrimp (Rengpipat et al., 2003) and Pacific white shrimp (Cai et al., 2019; Li, Tan & Mai, 2009b; Liu et al., 2014).

While the use of host-associated probiotics has drawn attentions as they improve effectiveness and sustainability of shellfish aquaculture (Doan et al., 2020), it is vital to understand the relationship between host shrimp and their intestinal microbiota in order to select potential probiotic candidates for the industry (Bentzon-Tilia, Sonnenschein & Gram, 2016; Stentiford et al., 2017). Recent advances in high-throughput omics-based technologies have provided crucial tools to further understand host-microbial interaction (Wang et al., 2019). Hence, to elucidate roles of shrimp gut microbiota and host responses in association with growth performance, multi -omics approach has been employed to explore bacterial compositions, gene expression and metabolomics profiles from the intestines of the black tiger shrimp. Intestine samples were collected from 5-month old juveniles of the same family, gender, diet and rearing conditions to identify intestinal microbiota, and host gene expression and metabolites associated with shrimp growth. The multi-omics analyses provide a global view of shrimp biological systems and insights into factors contributing to growth performance.

## Materials & Methods

### Ethics statement

All experimental protocols were approved by the animal ethical committee at the National Center for Genetic Engineering and Biotechnology (Approval code BT-Animal 04/2560) and carried out in accordance with the relevant guidelines and regulations.

### Shrimp rearing conditions and intestinal sample collection

Black tiger shrimp were reared and maintained at the Shrimp Genetic Improvement Center (SGIC), Surat Thani (Thailand). Five-month old juvenile female black tiger shrimp from the same family (*n* = 150) were harvested, individually weighted, and sorted based on their weight in ascending order. Shrimp with their weight above the 75th percentile and below 25th percentile were selected and called the Top-weight group and the Bottom-weight group, respectively. Average shrimp body weight of the Top-weight group (*n* = 35) was 36.6 ± 0.8 g, and the Bottom-weight group (*n* = 35) was 18.2 ± 1.8 g. Shrimp intestines were aseptically collected, and visible traces of fecal matter was gently removed by using sterile forceps. All intestine tissues were quickly frozen in liquid nitrogen and store at −80 °C until further used.

### 16S rDNA amplicon preparation and sequencing

DNA was extracted from each shrimp intestine using a QIAamp DNA extraction kit (Qiagen, USA) according to the manufacturer’s instruction (*n* = 15 for each weight group). All genomic DNA samples were cleaned up using a genomic DNA clean & concentrator™ (Zymo Research, USA). DNA concentration was determined by using a NanoDrop™ ND-8000 spectrophotometer (Thermo Fisher Scientific, USA). The V3 and V4 variable regions of the 16S rRNA genes were amplified by the following primer pair; forward primer (5′TCGTCGGCAGCGTCAGATGTGTATAAGAGACAGCCTACGGGNGGCWGCAG 3′) and reverse primer (5′GTCTCGTGGGCTCGGAGATGTGTATAAGAGACAGGA- CTACHVGGGTATCTAATCC 3′) for next-generation sequencing-based diversity analyses, in which Illumina overhang adapters were added to the gene-specific sequences. An amount of 100 ng of DNA sample was used as a PCR template to construct a 16S amplicon library with Q5 high-fidelity DNA polymerase (New England Biolabs Inc., USA). The PCR cycle parameters were an initial denaturation at 98 °C for 3 min, followed by 25 cycles of 98 °C denaturation for 30 s, a 57 °C annealing for 45 s, and a 72 °C extension for 30 s, and a final extension at 72 °C for 5 min. PCR reaction was performed in four replicates without reaching saturation to reduce biases. Each replicate was pooled together and purified by using QIAquick gel extraction kit (Qiagen, USA). All genomic DNA from each library were sent for 16S rRNA amplicon sequencing with Illumina MiSeq next-generation sequencing service (Macrogen Inc, Korea). Each DNA library (*n* = 30) was sequenced twice for technical replicates.

### RNA extraction and sequencing

Each shrimp intestine from the Bottom-weight group (*n* = 5) and the Top-weight group (*n* = 5) was ground with a pestle and mortar into fine power in liquid nitrogen and subjected to RNA extraction by using TriReagent^®^ (Molecular Research Center, USA) according to the manufacturer’s instruction. All RNA samples were treated with 0.5 unit/µg RQ1 RNase-free DNase (Promega, USA) for 30 min at 37 °C to remove DNA contamination, purified by using phenol:chloroform extraction and precipitated with isopropanol. The concentration of DNA-free RNA was measured using NanoDrop™ ND-8000 spectrophotometer (Thermo Fisher Scientific, USA). Integrity of total RNA was visualized under 1% agarose gel electrophoresis. Each RNA library was constructed by using TruSeq^®^ stranded mRNA LT sample preparation kit (Illumina Inc., USA) according to supplier’s instruction. All procedures for RNA sequencing analysis were conducted by Macrogen (Seoul, South Korea) using Illumina Hiseq platform (Illumina Inc., USA).

### Amplicon sequence analysis

Paired-end sequences of the 16S DNA amplicons were processed using SHiny application for Metagenomic Analysis (SHAMAN, http://shaman.c3bi.pasteur.fr/) (Quereda et al., 2016). Briefly, the raw sequencing reads were cleaned up by removing library adapters, primers and base pairs occurring at 5′ and 3′ ends with a Phred quality score < 20 using Alientrimmer (v0.4.0) (Criscuolo & Brisse, 2013). Paired-end reads were merged into an amplicon fragment using FLASH (Magoc & Salzberg, 2011). Chimeric sequences and singletons were removed by VSEARCH (Rognes et al., 2016). The sequences were clustered to Operational Taxonomic Units (OTUs) at a 97% sequence identity threshold using VSEARCH (Rognes et al., 2016). The taxonomy annotation was performed with SILVA v132 (Yilmaz et al., 2014). To identify taxonomical profiles at the species level, the representative sequences of OTUs were aligned with the NCBI 16S rRNA database using consensus BLAST with 98% sequence identity in Qiime2 (Hall & Beiko, 2018). The OTU sequences were aligned by using MAFF plugin (Katoh et al., 2002) and a phylogenetic tree was constructed by using FastTree2 plugin in Qiime2 (Price, Dehal & Arkin, 2010). Raw read counts are available in supplementary Table S1. Read count and representative sequences were exported and analyzed with the following programs. Rarefaction of all observed OTUs was performed for each library with a step size of 1 using rarecurve function in the vegan R package v2.5-6 (Oksanen et al., 2019). Diversity indices (Chao1, Shannon) and principal coordinates analysis (PCoA) were performed using Phyloseq (McMurdie & Holmes, 2013) in R 3.5.2 (R Core Team, 2019; RStudio Team, 2015). The microbiota dataset was normalized by the total number of reads in each sample to remove potential biases related to different sequencing depths (Md Zoqratt et al., 2018; Segata et al., 2011). *OTUs* with less than three sequences and not present in at least 12 samples of the total samples in a group were discarded for relative abundance analysis. Statistical significance of taxon abundance between groups was identified by using linear discriminant analysis (LDA) effect size (LEfSe) method (Segata et al., 2011) via the Galaxy platform (http://huttenhower.sph.harvard.edu/galaxy). A taxon with significant different abundance between the two weight groups was determined by LEfSe with Kruskal–Wallis rank sum test (*p* < 0.05) and LDA score (>2). The cladogram representing bacterial composition found in significant abundance of the Top-weight group and the Bottom-weight group was generated by using MEGAN (Huson et al., 2007).

### Co-occurrence analysis

Structure of microbial communities in each growth performance group was constructed using Co-occurrence Network inference (CoNet) (Faust & Raes, 2016) under Cytoscape v3.7 (Shannon et al., 2003). Network analysis of OTUs presented at > 50% in at least one study group was performed using Pearson and Spearman correlation (threshold 0.8) with Fisher z-transformation and Benjamini Hochberg testing (*p*-value < 0.05).

### RNAseq analysis

Sequencing reads were processed to remove adapter sequences, repetitive k-mers and low-quality sequences by FASTQC (Andrews, 2010) and MultiQC (Ewels et al., 2016). The raw reads were cleaned up by removing adaptor-sequences and low-quality bases at 3′end (Phread score < 20) using TrimGalore (https://github.com/FelixKrueger/TrimGalore). The filtered reads were assembled using Trinity (Grabherr et al., 2011). The assembled transcripts were evaluated by Universal Single-Copy Orthologs (BUSCO) with default settings and BUSCO v3.0.2 core dataset for single-copy conserved eukaryotic genes (Waterhouse et al., 2018). To avoid redundant transcripts, the longest transcripts of each gene based on Trinity assembly were selected and clustered using CD-HIT (Tassanakajon et al., 2006) with a 95% sequence identity cutoff and 80% minimal alignment coverage for the shorter sequence. The assembled sequences were annotated using FunctionAnnotator (Chen et al., 2017). Protein coding region of the non-redundant transcripts was determined using Transdecoder with default parameters (http://transdecoder.sourceforge.net). Gene ontology and Clusters of Orthologous Groups (COG) were assigned using eggnog-mapper (Huerta-Cepas et al., 2017) based on eggNOG 4.5 orthology data (Huerta-Cepas et al., 2016). The read counts were obtained by mapping the read to the non-redundant transcripts using Bowtie2 (Langmead & Salzberg, 2012) and SAMtools (Li et al., 2009a). Differently expressed genes (DEGs) were identified using the DESeq2 (Love, Huber & Anders, 2014). A size factor was used to normalize their library sizes and RNA composition biases (Love, Huber & Anders, 2014; Van den Berge et al., 2019). A multiple testing using the Benjamini–Hochberg multiple test was employed to select genes with a criterion of a greater than a two-fold change of the read counts from the Top-weight group and those from the Bottom-weight group with an adjusted *p*-value < 0.05. The enrichment of functional genes was employed using KOBAS 3.0 to further gain insights on transcriptional differences between the two shrimp groups (Xie et al., 2011).

### Validation of transcriptomic profiling

To validate the RNAseq results, quantitative real-time PCR (qPCR) were carried out to determine gene expression levels of the 10 selected genes. Gene-specific qPCR primers were designed by using Primer3 (Untergasser et al., 2012) (Table S2). Total RNA (1.5 µg) was reverse transcribed into cDNA using an ImPromII™ Reverse Transcriptase System kit (Promega, USA) according to the manufacturer’s recommendation. Each 20 µL qPCR reaction included 0.5 µL cDNA, 200 nM of each primer, and SsoAdvanced™ Universal SYBR^®^ Green supermix (Bio-Rad, USA) according to the company’s instruction. The thermal cycling parameters were 95 °C for 30 s, followed by 40 cycles of 95 °C for 15 s, 57 °C for 30 s and 72 °C for 30 s. The melting curve analysis was performed from 65 °C to 95 °C with a continuous fluorescent reading with a 0.5 °C increment. The threshold cycle (Ct) was analyzed using BioRad CFX Manager 2.1 software (Bio-Rad, USA).

### Extraction of shrimp intestine metabolites

Shrimp intestines (*n* = 15 for each weight group) were stripped off any visible fecal matters. Each intestine (∼100 mg fresh weight) was ground into fine powder using a ball mill grinder (MM400, Retsch), dissolved in one mL of cold ethyl acetate:acetone (15:2 v/v) extraction solvent acidified with 0.5% acetic acid and mixed at 2,400 rpm for 5 min using Multi-Tube vortexer before being sonicated at 4 °C for 15 min using Ultrasonic Cleaner (Crest D, USA). Samples were subsequently centrifuged at 4 °C, 10,000xg for 10 min to separate supernatant. The extraction step was performed twice, and the supernatants were pooled. The supernatant (two mL) was dried under vacuum at 35 °C for 20 min using a TurboVap nitrogen evaporator (Biotage, Sweden). Dried samples were re-dissolved by 150 µL of acetonitrile (Optima LC/MS grade) and filtered by micro centrifuge 0.22 µm PVDF membrane at 4 °C, 10000xg for 5 min. Each flow-through sample (100 µL) was transferred to an LC vial with a micro-insert prior to LC-HRMS injection. For reproducibility measurement of the instrument, pooled biological samples (n_pooled_ = 30) were used as quality control samples injected every 10 samples. In addition, sample without shrimp intestine was performed in parallel with the same extraction process and was used as a blank sample.

### Untargeted shrimp intestines metabolome using liquid chromatography-mass spectrometry

Untargeted metabolite profiles were carried out by using Dionex RS3000 in coupled with a Thermo Scientific™ Orbitrap Fusion™ Tribrid™ mass spectrometer. Reverse-phase chromatography was performed using HSS T3 C18 column (2.1 × 100 mm, 1.8 µm, Thermo), using mobile phase (Solvent A: water + 0.1% acetic acid and Solvent B: acetronitrile + 0.1% acetic acid) with gradient elution system as follows: an isocratic period for 2.00 min at 99:1 (A:B) was followed by a linear gradient to 1:99 (A:B) for 18.00 min, and hold for 5.00 min, followed by restoring initial conditions 99:1 (A:B) for 1.00 min and hold for 5.00 min.

Mass spectra were acquired using full scan data-dependent MS/MS under positive and negative electrospray ionization (ESI) modes over the mass range 50–1,200 Da. Ion source was performed using voltage of 3,500 V, ion transfer temperature of 325 °C and vaporizer temperature of 275 °C. The MS full scan resolution was set at 120,000 FWHM and MS2 scan resolution was set at 15,000 FWHM with 25% higher-energy collisional dissociations (HCD).

### Metabolite data processing and analyses

The acquired MS data of samples, blank sample and pooled samples were processed by Compound Discoverer (CD) 2.3.0 software using a workflow as follows: retention time alignment, unknown compound detection, elemental compositions prediction, chemical background subtraction using blank samples and compound identification using ChemSpider (Pence & Williams, 2010), HMDB (Wishart et al., 2007), KEGG (Kanehisa & Goto, 2000), LipidMAPS (formula or exact mass, http://www.lipidmaps.org/) and mzCloud (mass fragmentation pattern, http://www.mzcloud.org). Metabolite features annotated by aforementioned criteria were collected for further analysis. Partial least square-discriminant analysis (PLS-DA) was used to classify between Top-weight and Bottom-weight groups using SIMCA 15 (Umea, Sweden). Variable importance in projection (VIP) value in both positive and negative ESI modes greater than one was selected for clustergram analysis. The clustergrams were visualized to identify alteration patterns between Top-weight and Bottom-weight sample groups using cluster analysis by Cluster 3.0 software (Eisen et al., 1998). Metabolites features with greater than 1.5-fold change (Top-/Bottom-weight groups) were selected. Significant differences of the selected metabolite levels from the Top- and Bottom-weight groups were determined by paired sample *t*-test using Microsoft Excel 2013 software (Microsoft Windows).

### Hierarchical clustering of -omics and multi-omics

To correlate shrimp size and their -omics profiles, the hierarchical clustering analysis for each -omics were performed based on Spearman rank correlation by using hclust function in R (R Core Team, 2019). Each omics dataset was filtered to remove the noise variables (Bodein et al., 2019; Rappoport & Shamir, 2018). Briefly, the microbiome dataset was normalized by the total number of reads in each sample to remove potential biases related to different sequencing depths (Md Zoqratt et al., 2018; Segata et al., 2011). OTUs with less than three sequences and not present in at least 12 samples of the total samples in a group were discarded. Nonparametric factorial Kruskal-Wallis (KW) rank sum test was first employed to examine whether OTUs abundance in weight groups was differentially distributed. The linear discriminant analysis effect size (LEfSe) was used to access discriminatory power of each OTUs (Segata et al., 2011). For transcriptomic data, size factor was used to normalize their library sizes and RNA composition biases (Love, Huber & Anders, 2014; Van den Berge et al., 2019). The normalization factor was calculated by taking the median of the ratio between read counts and geometric mean derived from each gene across samples. Dispersion parameters in negative binomial distribution were estimated by the Bayesian shrinkage approach to model gene counts. Finally, the Wald test was used to select genes (Love, Huber & Anders, 2014). For metabolomics, chromatographic peak area of each metabolite from all samples was normalized to the total area of the corresponding samples to adjust their differences in intensity values (Kapoore & Vaidyanathan, 2016). Partial least squared discriminant analysis (PLS-DA) was performed to filter metabolites that were significantly found in shrimp. Variable importance in projections coefficients obtained during the PLS-DA was set as a threshold value of 1, therefore, metabolites with their VIP > 1 were selected. Therefore, 53 most abundant OTUs were obtained for microbial profiles, 444 genes were filtered from transcriptomics and 90 metabolites were obtained from metabolomics profiles (Table S3). To generate integrative clustering analysis, the equal sampling depth was required. Thus, random sampling was employed to adjust sample size equal to 10 for each -omics before integrating multi-omics for clustering analysis. The cluster trees from randomized samples were aggregated to generate a consensus tree using MergeTrees (Hulot et al., 2020).

## Results

### Sequencing depth and intestinal microbiota profiles

The intestines collected from the 5-month-old black tiger shrimp juveniles from the Top-weight group and the Bottom-weight group were subjected to microbiota analysis using paired-end next-generation sequencing of V3 and V4 regions on the 16S rRNA genes. An average of 137,512 sequences per sample and a range of 98,465-165,593 sequences were obtained after quality filtering and chimera removal. Alpha diversity indices including Shannon and Chao1 showed no significant differences (*p*-value > 0.05) between the Top-weight group and the Bottom-weight group (Table S5), suggesting for similar levels of bacterial diversity. Additionally, Chao1 estimated 298 ± 50 and 319 ± 53 OTUs for the microbiota in the Top-weight and Bottom-weight groups, respectively, in which the 253 ± 47 OTUs and 271 ± 51 OTUs were observed in the Top-and Bottom-weight shrimp, respectively. The higher values of Chao1 (*p*-value < 0.05, *t*-test) suggests that more species is expected to be found with higher sequencing depth, which was consistent to the unsaturation of richness in rarefaction analysis (Fig. S1). The overall bacterial relative abundance was similar among the Top-weight and the Bottom-weight groups (Fig. S2 and Table S1) with similar dominant phyla including Proteobacteria, Fusobacteria, and Bacteroidetes (Fig. S2A). However, the frequency distribution of bacterial phyla slightly differed between the two groups. The individual variation of microbiota profiles was observed at the genus level (Fig. S2B). For instance, *Pseudoalteromonas* was found in higher abundance (> 5%) in three shrimp from the Bottom-weight group.

**Figure 1:**
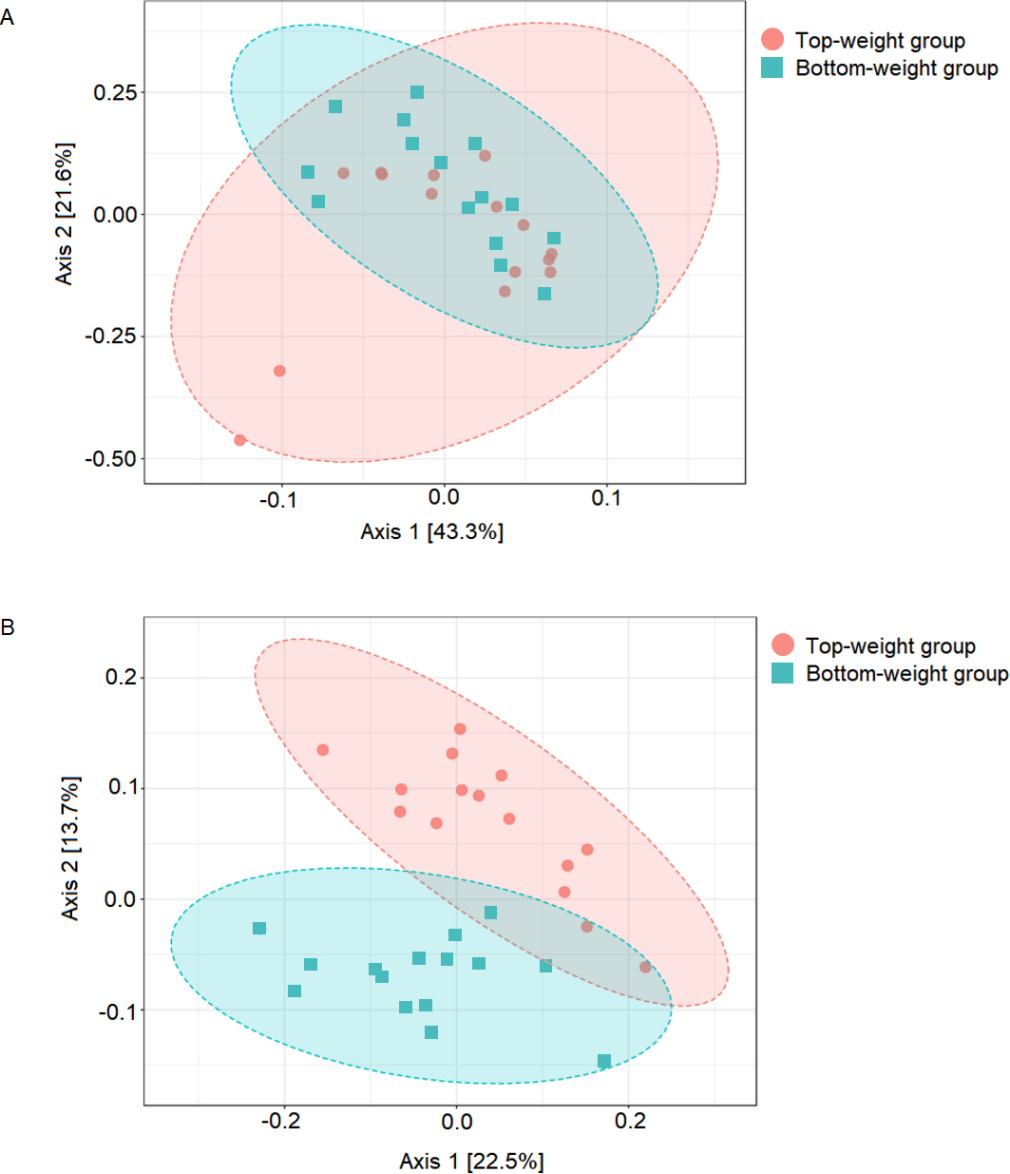
Comparison of shrimp intestinal microbial communities between Top-weight group (*n* = 15) and Bottom weight group (*n* = 15). The PCoA plot was generated using (A) weighted-UniFrac analysis (PERMANOVA *F*-value 1.84, *p*-value <0.12) and (B) unweighted-UniFrac analysis (PERMANOVA *F*-value 4.02 *p*-value < 0.001). The red represents intestinal bacterial profiles associated with the Top-weight group and blue represent bacterial profiles associated with the Bottom-weight group.

**Figure 2:**
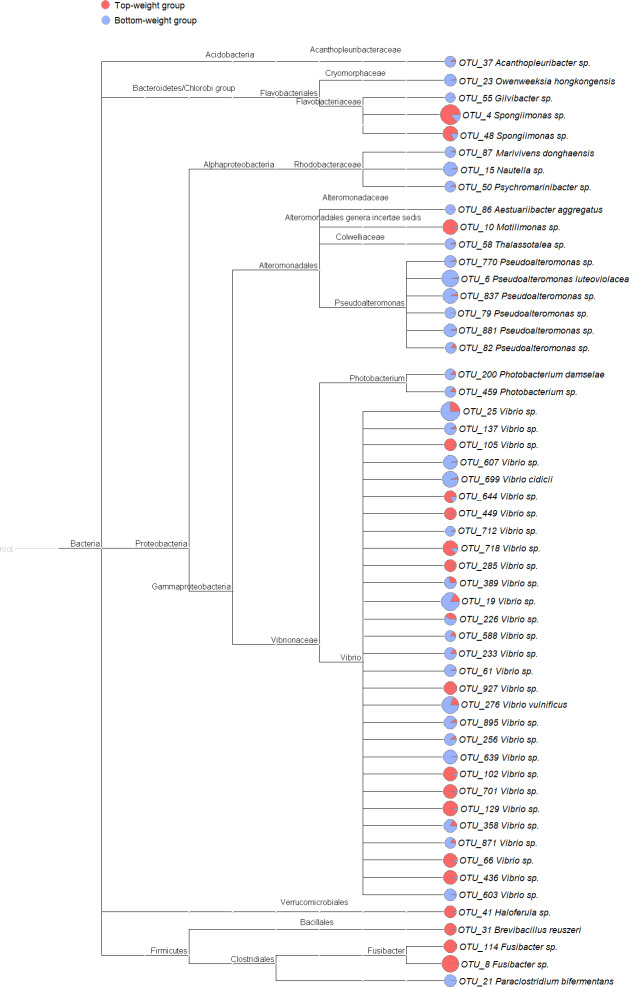
Cladogram showing differential relative abundance of 53 OTUs-level taxa between the Top-weight group and the Bottom-weight group. The significantly differentially abundant OTUs were identified by linear discriminant analysis (LDA) combined with effect size measurements (LEfSe). The cutoff criteria were *p*-value < 0.05, false discovery rate < 0.05 and the logarithm of LDA score >2. The pie chart depicts relative abundance of each genus found in the Top-weight group (red) or in the Bottom-weight group (blue) in log scale.

### Comparison of bacterial community structures in shrimp intestines from the Top-weight and the Bottom-weight groups

UniFrac-based principal coordinates analysis (PCoA) was applied to compare bacterial communities of all samples in the Top-weight and Bottom-weight groups (Fig. 1). Weighted-UniFrac-based PCoA analysis showed that bacteria associated to the Top-weight group and the Bottom-weight group were mostly overlapped (Fig. 1A), suggesting that overall relative bacterial abundant profiles were quite similar in both groups. Permutation multivariate analysis of variance (*PERMANOVA*) confirmed that there was no statistical significance (*p*-value = 0.12) between both groups. On the other hand, unweighted-UniFrac-based PCoA, (Fig. 1B) comparing a presence or absence of a bacterium showed distinct clusters between the Top-weight group and the Bottom-weight group (*p*-value < 0.001), suggesting that unique bacterial taxa associated with either weight group contributing to differences in community structures.

To identify potential growth-related bacteria, the linear discrimination analysis (LDA) using LEfSe was employed to identify significant bacterial taxa using a threshold of LDA score higher than 2. There was a total of 53 significant OTUs, of which 18 OTUs were found to be significantly more abundant in the Top-weight group (Fig. 2A). Conversely, the 35 OTUs were significantly present in the Bottom-weight group. The OTUs were classified into three phyla and seven genera. At the phylum level, *Acidobacteria* was significantly more abundant in the Bottom-weight group, while *Verrucomicrobia* and *Firmicutes* were significantly more abundant in the Top-weight group. At a genus level, *Spongiimonas*, *Haloferula*, *Brevibacillus*, and *Fusibacer* were found in a significantly higher in the Top-weight group, whereas abundance of *Owenweeksia*, *Givibacter*, *Nautella* were significantly higher in the Bottom-weight group. Among those 53 OTUs, only 8 OTUs could be assigned to species level, namely, *Vibrio vulnificus* (OTU_276), *V. cidicii* (OTU_699), *Marivivens donghaensis* (OTU_87), *Aestuariibacter aggregatus* (OTU_86), *Pseudoalteromonas luteoviolacea* (OTU_6), *Photobacterium damselae* (OTU_200), *Paraclostridium bifermentans* (OTU_21) and *Brevibacillus reuszeri* (OTU_31). All except *B. reuszeri (OTU_31)* were associated in a high abundance with the Bottom-weight shrimp. The genus *Vibrio* was most prevalent OTUs (29 out of 53 significant OTUs).

**Figure 3:**
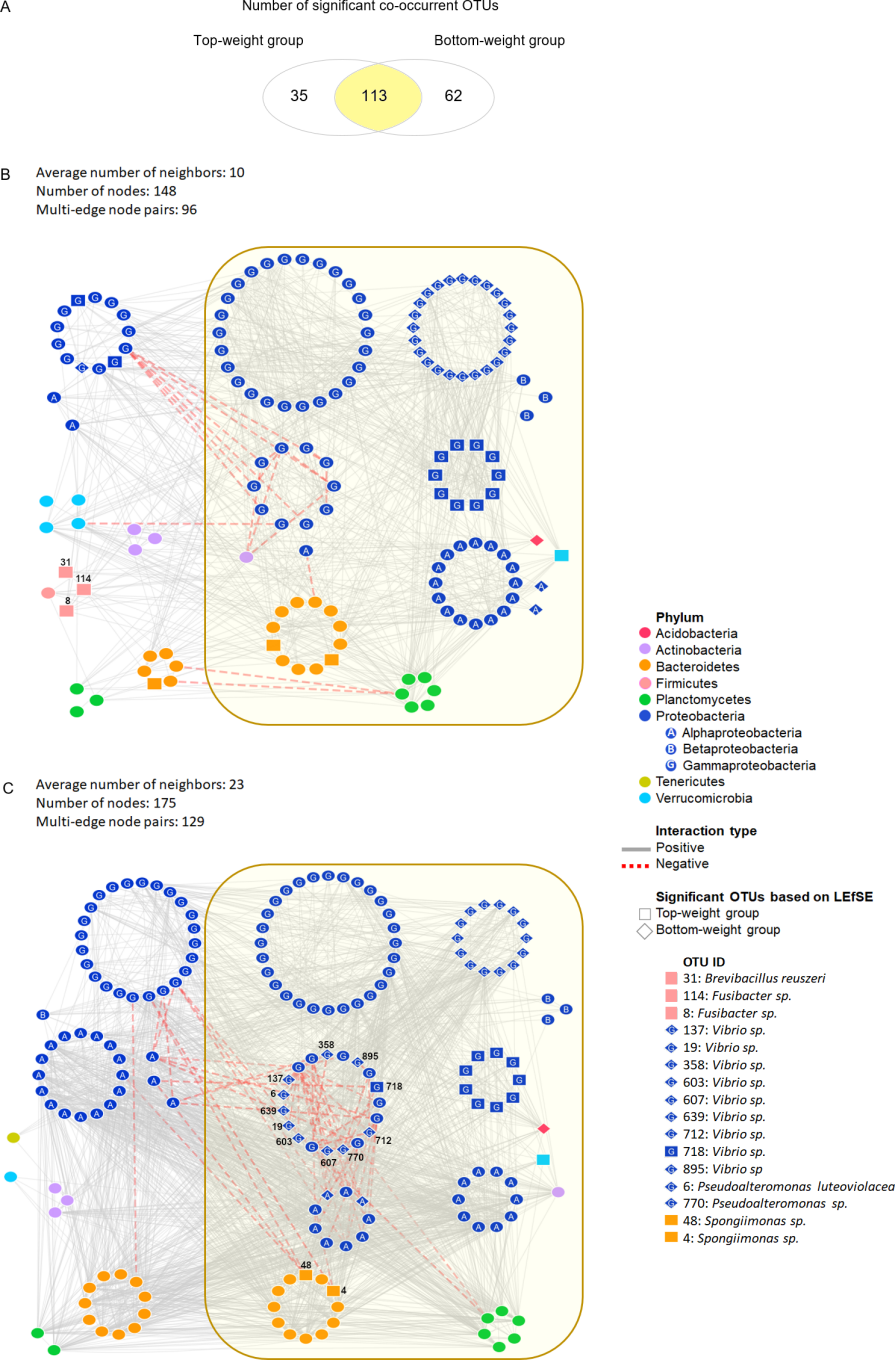
Co-occurrence networks of bacterial community in the Top-weight group and the Bottom-weight group. Venn diagram (A) showing a number of significant co-occurrent OTUs between the Top-weight group and Bottom-weight group. Bacterial interaction network in the Top-weight group (B) and Bottom-weight group (C) were generated in CoNet and visualized in Cytoscape to display significant strong positive (grey line) and negative (red line) co-occurring relationships. Nodes correspond to microbial taxa (OTU) and each taxonomy is illustrated in different color. Diamond and rectangular shapes represent significant OTUs identified by LEfSe.

### Co-occurrence analysis of bacterial network in the intestines of Top-weight and Bottom-weight groups

We assessed the network model of the microbial communities in each growth performance group using Co-occurrence Network inference (CoNet) (Fig. 3). Comparisons among taxa from both networks showed that 113 nodes were shared between the two weight groups, whereas 35 and 62 nodes were unique to the Top-weight group and Bottom-weight group, respectively (Fig. 3A). Hence, a large proportion of the bacterial co-occurrence networks for both groups contained similar taxonomic distribution (Figs. 3B and 3C), with *Proteobacteria* dominating in the networks. Although OTUs were mostly shared in both bacterial networks, the higher number of negative correlations (e.g., competitive association) was observed in the Bottom-weight group than in the Top-weight group (Figs. 3B and 3C), indicating a higher competitive interaction within their microbial community. For instance, a group of *Vibrio spp.* OTUs (OTUs: 137, 607, 712, 718, 19, 895, 639, 358, and 603) showed negative co-occurrence in the Bottom-weight shrimp (Fig. 3C) suggesting competitive interactions among these OTUs. *Spongiimonas spp.* (OTUs: 4 and 48), which their abundance significantly decreased in the Bottom-weight group, were negatively correlated to other Proteobacteria in the Bottom-weight group (Fig. 3C). Our co-occurrence network analysis provides an evidence revealing that interactions of intestinal bacteria were different in shrimp with different growth performance. Among the OTUs found associated in the Top-weight group (Fig. 3B), three Firmicutes OTUs (OTUs: 8, 31 and 114) belong to *Brevibacillus sp.* (OTU 31) and *Fusibacter sp*. (OTUs: 8 and 114) were unique to this group.

### Transcriptomic profiling in shrimp intestines under different growth performance

**Figure 4:**
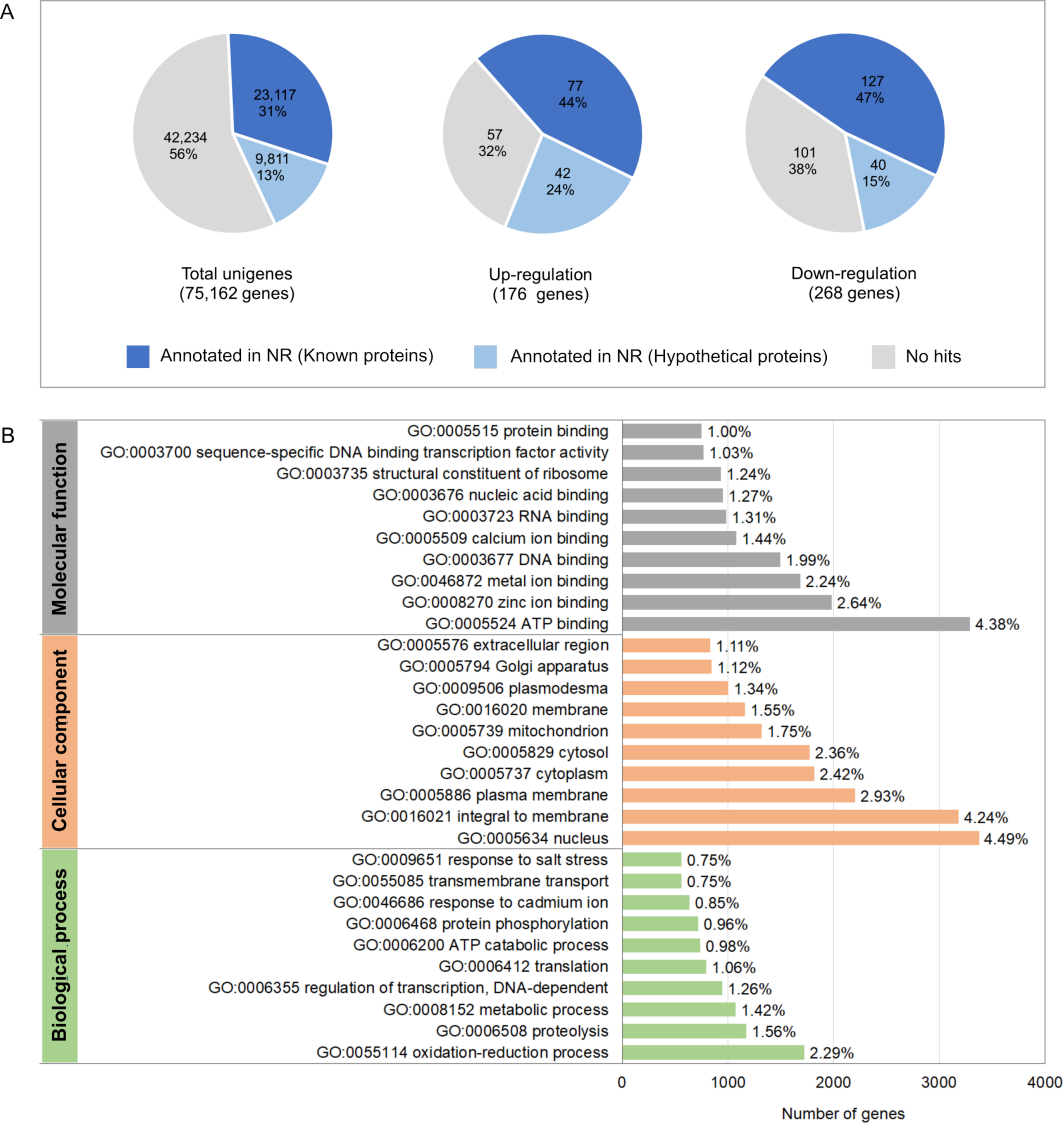
Annotation summary of transcripts in intestines of the Top-weight group and Bottom-weight group. (A) Annotation of unigenes and significant differentially expressed genes using BLAST against NCBI non-redundant protein database (NR). The pie chart shows the percentage of the genes matched sequences in the database. (B) Histogram of gene ontology of the unigenes which were classified into three main categories: biological process, cellular component, and molecular function.

To investigate molecular mechanisms in relation to growth performance, transcriptomics analysis was carried in intestines from the Top-weight group and the Bottom-weight group. Paired-end Illumina sequences were generated from ten individual biological samples from the Top-weight group (*n* = 5) and the Bottom-weight group (*n* = 5). A total of 50,258,288,995 nucleotides and 363,229,646 reads were obtained for downstream analysis. Due to the unavailable reference genome of the black tiger shrimp, *de novo* assembly using Trinity (Grabherr et al., 2011) was employed, and 75,162 unigenes i.e., non-redundant assembled genes were generated. The transcriptome completeness and quality of *de novo* assembled sequences were evaluated using BUSCO, which 84.2% of our transcriptome (77.6% of single copy and 6.6% of duplicated) were mapped. A few transcripts were fragmented (4.6%) and missing (11.2%). Overall ∼91% of reads were mapped back to unigenes, indicating high quality of transcriptome assembly for downstream analysis. Annotations were based on mapping information against various databases including NCBI non-redundant protein database (NR: 43.8%), *Kyoto* Encyclopedia of Genes and Genomes (KEGG: 19.6%), SwissProt (24.9%), PFAM (19.6%), Gene Ontology (GO: 28.7%) and eggNOG (46.8%) (Table S7). Based on the mapping to at least one database, 46.8% of the unigenes could be assigned to the functional profile. Since the black tiger shrimp genome is not yet available, the higher proportion of the un-assigned functional profiles to transcripts in our study was expected. Here, we defined differentially expressed transcript as those with absolute log_2_ fold-change > 1 and an adjusted *p*-value < 0.05. Fold-change was determined by taking a ratio of the Top-weight group over the Bottom-weight group. According to the defined threshold, 176 transcripts were up-regulated, and 268 transcripts were down-regulated (Fig. 4A and Table S7). Among those differentially expressed genes (DEGs), 44% of up-regulated genes and 47% of down-regulated genes showed homology in the NR database. GO terms corresponding to annotated genes were categories into three groups of biological process, cellular component, and molecular function (Fig. 4B). Top GO terms under molecular function were ATP binding (4.38%), zinc ion binding (2.64%) and metal ion binding (2.24%). The GO term of the cellular component was mostly classified to the nucleus (4.49%), integral to membrane (4.24%) and plasma membrane (2.93%). The most prevalence GO terms in the biological process were an oxidation–reduction process (2.29%), proteolysis (1.56%) and metabolic process (1.42%). The GO terms indicated that our RNA sequencing analysis cover comprehensive expression profiles for further pathway analysis.

**Figure 5:**
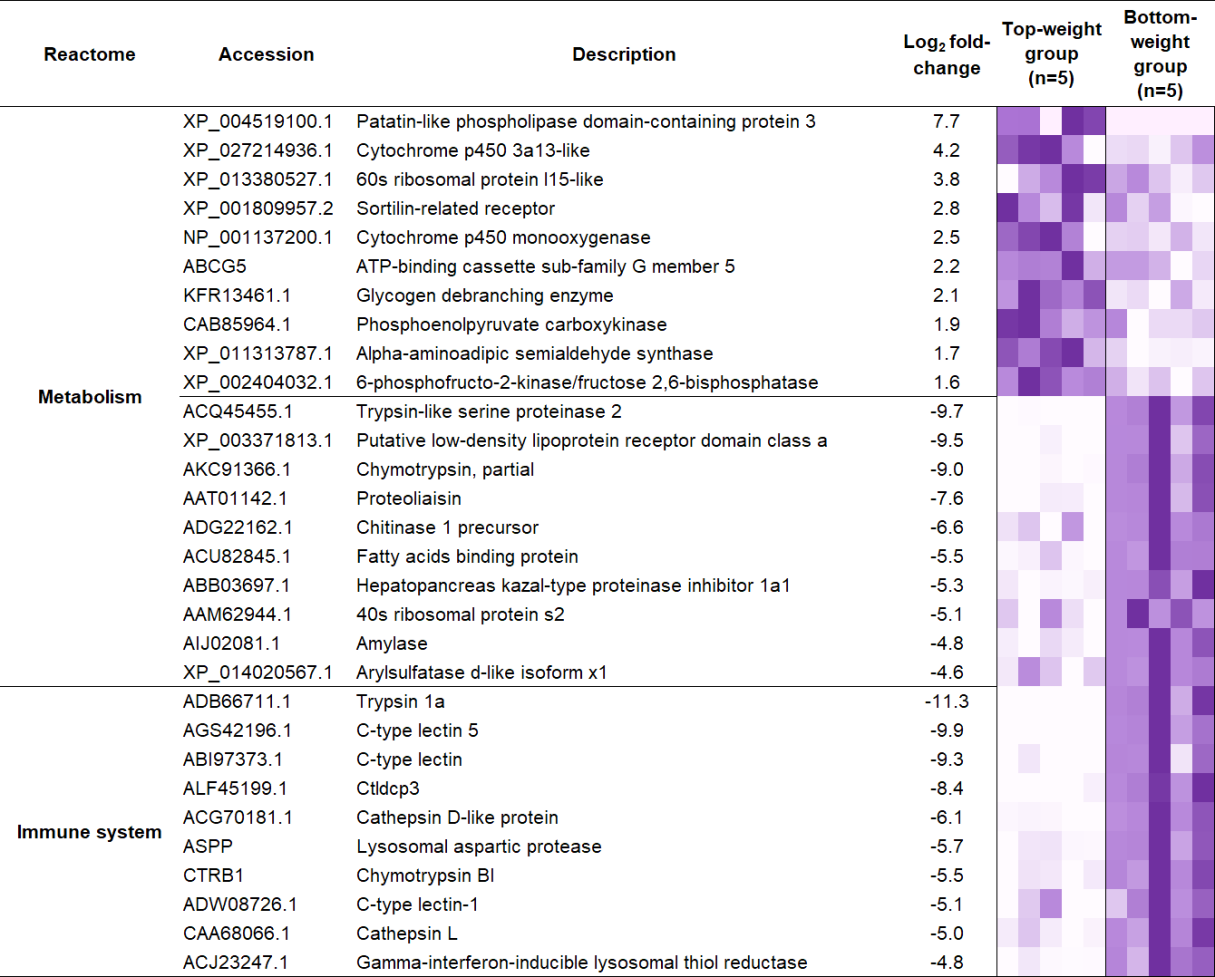
Heatmap representing top ten differentially expressed genes enriched in two main biological categories (i) metabolic and (ii) immune pathways. .

To understand the regulatory network of DEGs, statistical enrichment of the DEGs based on reactome database was carried out by using KOBAS. Reactome database is a curated collection of human molecular reactions and pathways which support pathway annotation of other organisms through orthology-based inference of pathways. A heatmap representing DEG profiles revealed gene patterns associated with the Top- or the Bottom-weight shrimp (Fig. 5). The top five enriched pathways of up-regulated and down-regulated genes were relevant to metabolism, developmental biology, glucose, carbohydrate and protein metabolisms and immune system (Table 1). Metabolism was commonly enriched in both groups. Notably, the enriched pathways in Bottom-weight group were also those involved in immune responses, suggesting that the Bottom-weight group might require a larger energy expenditure on immune responses, whereas genes in Top-weight group could effectively apply toward their growth development. In the Top-weight group, their top ten significantly upregulated genes involved in metabolism were mostly those involved in nutrient and energy through catabolic processes including lipolysis, glycolysis, and amino acid catabolism (Fig. 5). For instance, patatin-like phospholipase domain-containing protein 2 involves in energy homeostasis and storage through lipolysis (Yang & Mottillo, 2020). Glycogen debranching enzyme plays role in glycogen breakdown (Zhai et al., 2016). Phosphoenolpyruvate carboxykinase plays role in phosphoenolpyruvate synthesis for gluconeogenesis (Yang, Kalhan & Hanson, 2009). Moreover, alpha-aminoadipic semialdehyde synthase involves in lysine catabolism (Crowther et al., 2019) and 6-phosphofructo-2-kinase/fructose 2,6-bisphosphatase plays role in glycolysis pathway (Rider et al., 2004). In contrast, the top ten genes enriched in metabolism pathway of the Bottom-weight group involved in the breakdown of complex molecules such as trypsin-like serine proteinase 2, chymotrypsin, chitinase1, and amylase. Moreover, the metabolic genes found highly expressed in the Bottom-weight group could also play roles in immunological processes such as trypsin and serine proteinase inhibitors (Visetnan et al., 2009). Additionally, the significantly upregulated genes in the Bottom-weight group were trypsin and chymotrypsin (Patel, 2017), cathepsin (Latz, 2010), C-type lectins (Li et al., 2014), lysosomal aspartic protease and gamma-interferon-inducible lysosomal thiol reductase (Kongton et al., 2011), which have been well reported for their roles as a part of shrimp immune responses. Over-activation of the immune system under the normal growth conditions was observed in the Bottom-weight shrimp, providing a sign of stresses and this imbalance immune responses could influence their growth performance.

**Table 1:** Reactome pathway analysis of genes up-regulated and down-regulated in the Top-weight group when compared to the Bottom-weight group (corrected *p*-value < 0.05).

**Differential expression**	**Reactome ID**	**Pathway description**	**Number of genes**	**Corrected*p*-value**
Up-regulated	R-HSA-1430728	Metabolism	11	1.6E−03
	R-HSA-74160	Transcription, translation and the regulation of these processes	8	1.9E−02
	R-HSA-1266738	Developmental biology	6	9.1E−03
	R-HSA-70326	Glucose metabolism	3	2.1E−03
	R-HSA-71387	Carbohydrate metabolism	3	3.3E−02
Down-regulated	R-HSA-1430728	Metabolism	33	2.6E−15
	R-HSA-168256	Immune System	19	1.2E−06
	R-HSA-392499	Protein metabolism	14	2.3E−04
	R-HSA-168249	Innate immune system	12	2.3E−05
	R-HSA-1474244	Extracellular matrix organization	11	2.4E−08

### Metabolomics profiles analysis in intestines of the Top-weight and Bottom-weight shrimp

**Figure 6:**
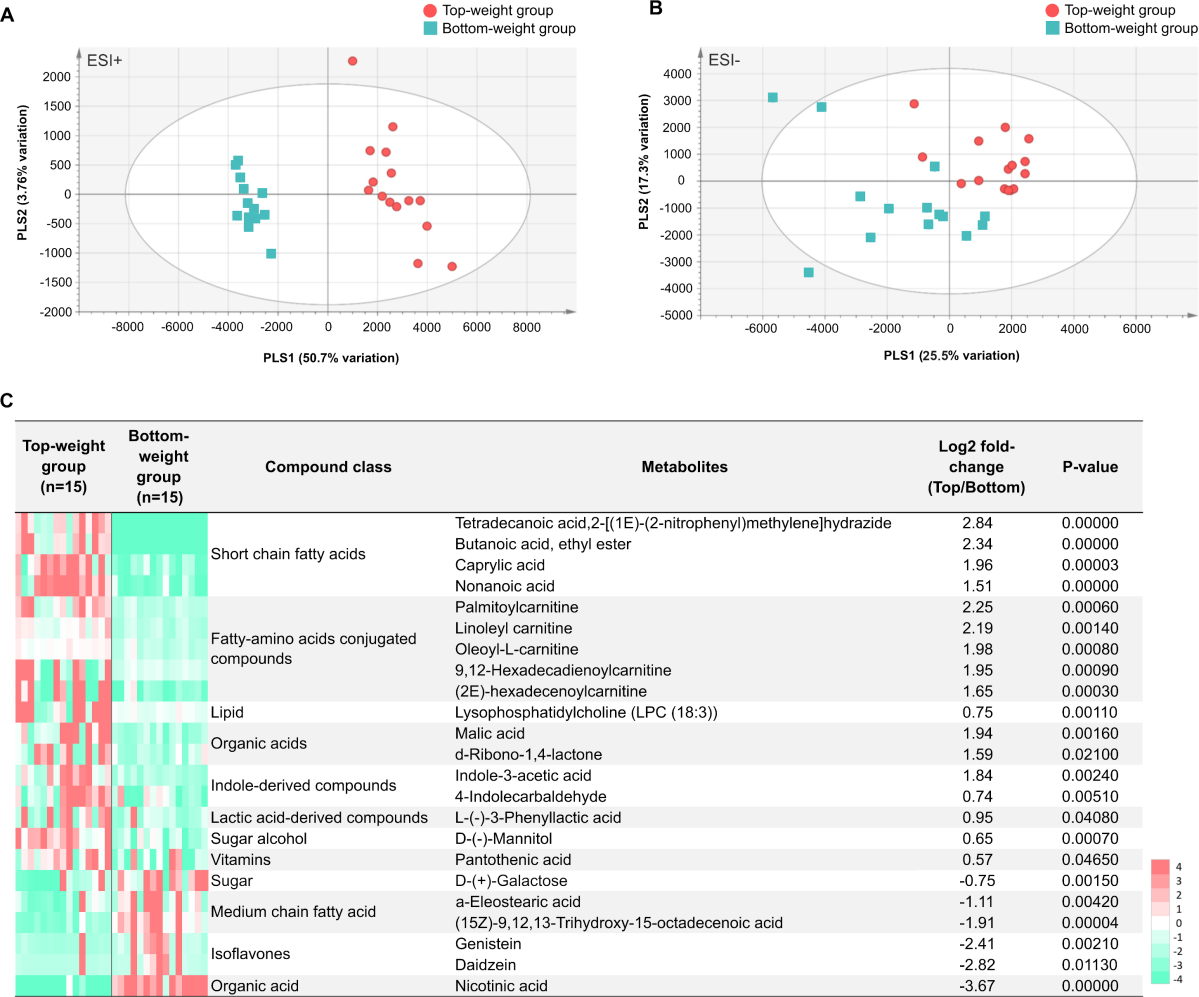
Metabolomics profiles analysis in intestines of the Top-weight and Bottom-weight shrimp. Principle component analysis of metabolome data obtained from (A) positive electrospray ionization (ESI+) and (B) negative electrospray ionization (ESI-) of the Top-weigh group (red) and the Bottom-weight group (green). (C) Heatmap of selected metabolites based on variable importance in projection (VIP) score were grouped based on their biochemical categories. *P*-value indicates statistical significance of the selected metabolite levels between the Top- and Bottom-weight groups.

The MS processed data between the Top- and the Bottom-weight groups revealed 1,044 ions for ESI+ mode and 360 ions for ESI- mode (Figs. 6A and 6B). These metabolite features were then filtered by selection of 90% annotation confidence based on matching scores in both molecular mass and their particular MS fragmentation patterns. Of these, 265 and 142 metabolite features were obtained from ESI+ and ESI- modes, respectively. The metabolite features were then subjected to partial lease square-discriminant analysis (PLS-DA) for visualization of global metabolic profiles between the sample groups. PLS-DA scores plot revealed distinctive differences between the Top- and the Bottom-weight groups in ESI+ with 50.7% variation in PLS1 and 3.7% variation in PLS2, whereas those in ESI- showed slightly different with 25.5% variation in PLS1 and 17.3% variation in PLS2 (Figs. 6A and 6B). The results suggested that the different metabolic profiles reflected different growth performance.

To select significant metabolites contributing to different growth performance, variable importance in projection (VIP) values was employed and identified a total of 58 and 32 metabolite features (VIP>1) in ESI+ and ESI-, respectively (Table S8). The intestines of the Top-weight shrimp contained significant higher levels (*p*-value < 0.05) of short chain fatty acids (butanoic acid), medium chain fatty acid (caprylic acid), indole-derived compounds (e.g., indole-3-acetic acid and 4-indolecarbaldehyde), lactic acid-derived compound (e.g., L-3-phenyllactic acid), fatty-amino acids conjugated compounds (palmitoylcarnitine, linoleyl carnitine and oleoyl-L-carnitine), organic acid (malic acid), sugar alcohol (e.g., mannitol), vitamin (pantothenic acid; vitamin B5) and lipid (e.g., lysophosphatidylcholine (LPC 18:3)) (Fig. 6C). On the other hand, the intestines of the Bottom-weight shrimp showed significant higher levels of galactose, isoflavones (genistein, daidzein) and nicotinic acid (Fig. 6C).

### Integrative clustering of multi-omics profiling to identify correlation to shrimp growth

**Figure 7:**
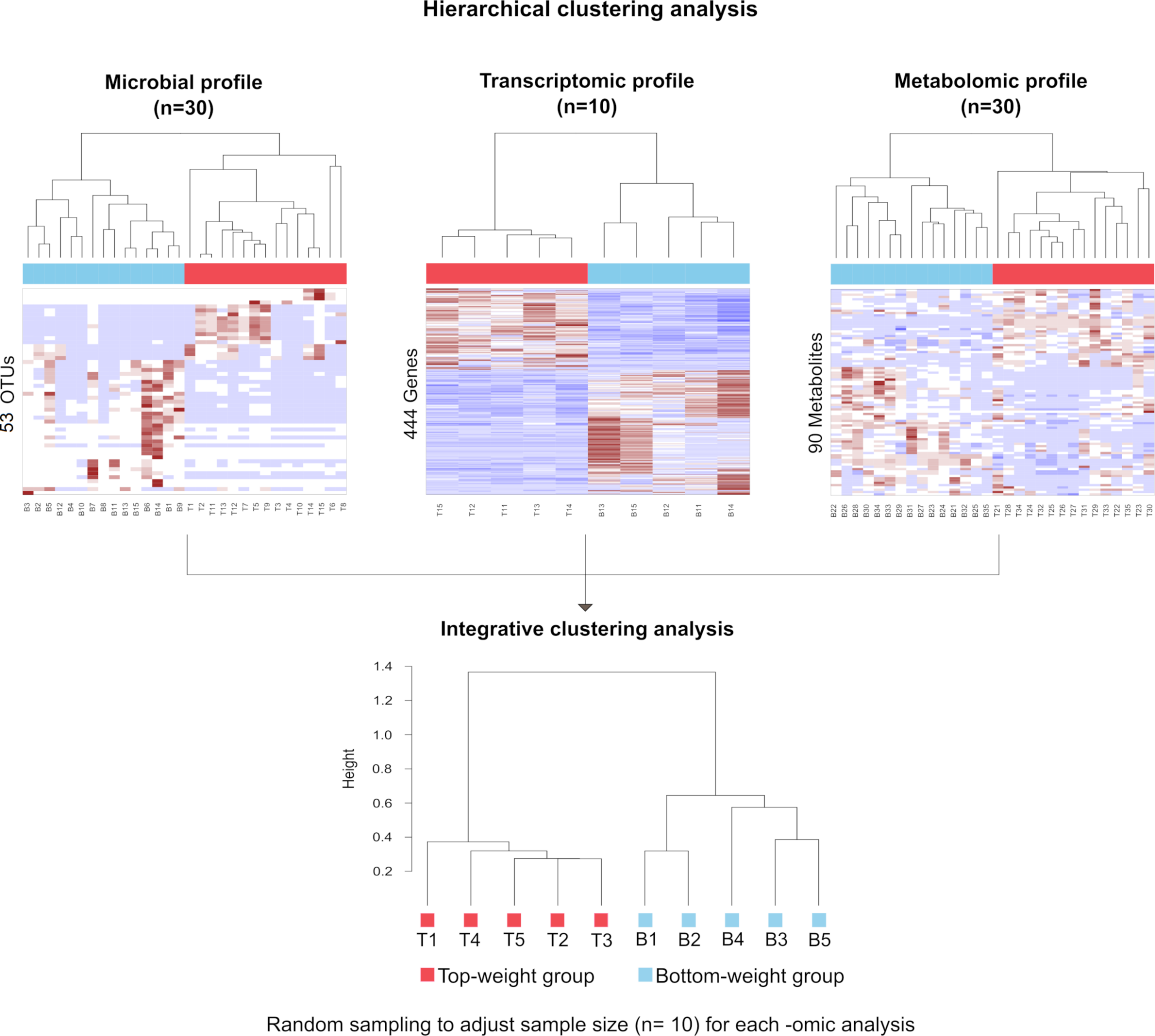
Hierarchical clustering analysis revealed bacteria, genes and metabolites correlated to shrimp weight. Selected microbial profiles (53 OTUs), transcriptomic profiles (444 genes) and metabolomic profiles (90 metabolites) were clustered using Spearmanrank correlation with average linkage. Integrative cluster analysis was employed to combine each -omic dataset to generate a consensus tree. Colors at the leaf nodes correspond to the shrimp weight group, in which red represents the Top-weight group and blue represents the Bottom-weight group.

**Figure 8:**
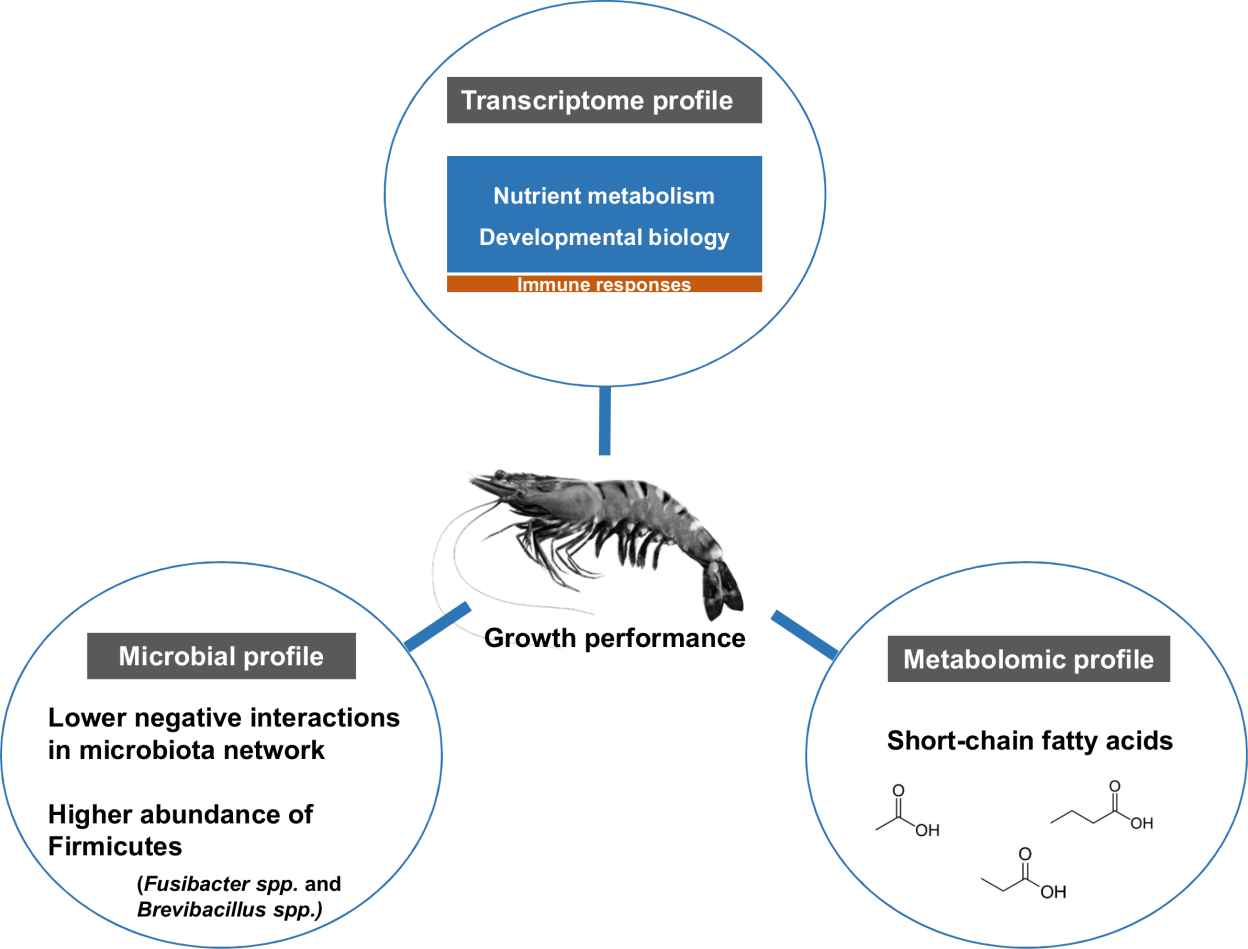
Factors contributing to shrimp growth performance.

The influence of shrimp size on all three omics was further explored from filtered features (53 most abundant bacterial OTUs, 444 differentially expressed genes and 90 metabolites) (Table S3) using hierarchical clustering (Fig. 7). The clustering analysis for each -omics profile revealed two major clusters based on shrimp weight group (Fig. 7), suggesting a correlation between shrimp sizes to their -omics profiles. Consistently, the integrative clustering analysis of multi-omics also showed two groups based on shrimp weight. Hence, the conceptual integration of these multi-omics profiles could infer importance of microbiota, host gene expression and metabolites contributing to growth (Fig. 8). Our integrative clustering of multi-omics revealed that shrimp growth performance was correlated to different abundance of Gram-positive bacteria, mainly Firmicutes. Gene expression profiles of the Top-weight shrimp showed upregulation of gene-related to nutrient metabolisms, while immune response genes were significantly upregulated in the Bottom-weight shrimp. Metabolomics analysis showed higher concentration of short-chain fatty acids in the intestines of the Top-weight shrimp.

## Discussion

Understanding the factors that contribute to shrimp growth performance will greatly enable us to decipher biological mechanisms, beneficial intestinal microbiota and approaches to optimize shrimp production through probiotics and prebiotics applications. Here, we integrated multi-omics analyses to determine how microbiota, host gene responses, and metabolites correlated to shrimp growth. The bacterial compositions were analyzed in parallel with host gene expression and metabolomics in shrimp intestines from different sizes. Shrimp from the same genetic background, rearing environments and diets were used in this study to minimize the effects of intrinsic and extrinsic factors. Shrimp were grouped based on their weight ranges in which the Top-weight group were shrimp with their weight twice bigger than the Bottom-weight group.

Dominant phyla (relative abundance > 1%) shared between the Top- and the Bottom- weight shrimp were Proteobacteria, Fusobacteria, and Bacteroidetes. Although the percent relative abundance were varied, the dominant phyla were consistent with the previous reports on intestinal bacterial profiles in black tiger shrimp (Rungrassamee et al., 2014) and Pacific white shrimp (Fan et al., 2019). The overall bacterial community structures were mostly similar in the two groups due to similar genetic background of the host and the same rearing conditions (Cornejo-Granados et al., 2018). However, some differences in key taxa might be relevant to growth performance. For instance, although OTU composition of the *Vibrio* genus were the similar between the two groups, relative abundance of 29 OTUs with in the genus were significantly different. *Vibrio* genus is commonly found associated to intestines of many aquatic organisms including shrimp (Md Zoqratt et al., 2018; Mongkol et al., 2018). This genus is highly diverse and contain pathogenic and non-pathogenic members, in which some have been reported to play beneficial roles to their animal host (Ceccarelli & Colwell, 2014; Thompson et al., 2010). Here, we speculate that highly enriched *Vibrio* species in the Top-weight group might provide growth-related benefits to host shrimp similar to *V. alginolyticus*, *V. salmonicida*, and *V. gazogenes*, which have been previously reported (Thompson et al., 2010; Verschuere et al., 2000). Further studies to obtain longer sequencing reads would be necessary to identify at the species level and to determine which *Vibrio* species could negatively or positively affect to shrimp growth.

Firmicutes, the third dominant phylum, was found in higher relative abundance in the Top-weight group (1.54 ± 3.93%) than the Bottom-weight group (0.13 ±  0.41%). Influence of Firmicutes in the gastrointestinal tract to body weight has gained more attention (Zhu et al., 2019). Firmicutes contribute to energy harvest from food by reportedly playing roles in the decomposition of polysaccharides (Turnbaugh et al., 2006). Our observation on association of Firmicutes to the Top-weight shrimp was consistent to the study in Pacific white shrimp (Fan & Li, 2019). Firmicutes is one of the important bacterial groups for health and immunity in crustaceans (Duan et al., 2018; Foysal et al., 2019). Although many studies have reported on the ratio of Firmicutes/Bacteroidetes ratio in correlation to growth performance (Fan & Li, 2019; Kasai et al., 2015; Li et al., 2013), the Firmicutes/Bacteroidetes ratio was not significantly different in this study.

The significant genera in Firmicutes found associated to the Top-weight group were *Brevibacillus, Fusibacter,* and *ParaclostridiumI.* OTU_31 (*Brevibacillus reuszeri*), OTU_8 (*Fusibacter spp.*) and OTU_114 (*Fusibacter spp.*). Particularly, several *Brevibacillus* spp. have been identified as potential probiotics in seabass (Mahdhi et al., 2012) and as antimicrobial peptide producers (Yang & Yousef, 2018). For example, *B. laterosporus* isolated from the honeybee digestive tract has been reported to have probiotic effect and promote growth of the honeybees (Khaled et al., 2018). *B. reuszeri* has been shown to be able to produce amino acids, which are essential nutrients required for growth (Wang et al., 2015). Therefore, *Brevibacillus*, which found associated with the Top-weight shrimp group, might be a promising candidate for probiotics in shrimp production. For instance, *B. brevis* has been applied as probiotic in swine production (Che et al., 2016).

Despite the different bacterial community interactions observed in the Top- and Bottom- weight groups, positive correlations dominated species-species networks in both groups, suggesting that higher cooperative bacterial interactions were observed in shrimp intestines (Williams, Howe & Hofmockel, 2014). Most negative interactions were related to Gammaproteobacteria in both shrimp groups. However, the higher number of negative correlations in the Bottom-weight group were observed, revealing mutual exclusion and higher bacterial competition than the Top-weight group. The negative correlation between different taxon as found in the Bottom-weight group could be due to direct inhibition, toxin production and bacterial adaptation (Dunny, Brickman & Dworkin, 2008). Higher negative bacterial correlations could also occur in an animal host trying to maintain its intestinal bacterial balance under disturbed conditions (Dai et al., 2018). Hence, the observation of higher negative correlations in the Bottom-weight shrimp could be an indicator of stresses, resulting in a lower growth performance. The network also revealed candidate OTUs for better growth performance i.e., *Brevibacillus reuszeri* (OTU_31), *Fusibacter sp.* (OTU_8 and OTU_31).

A number of genes involved in anti-bacterial host defense like phagosome and lysosome were highly expressed in the Bottom-weight group compared to that of the Top-weight group. Stimulation of immune response corresponded with several opportunistic bacteria observed in the Bottom-weight group. Immune function could be energetically costly, thus physical growth of an animal could be compromised when immune responses were stimulated (Cabrera-Martínez, Herrera & Cruz-Neto, 2018; Doeschl-Wilson et al., 2009). Growth is a result of energy balance between energy intake and energy expenditure (Talbot et al., 2018). As a result of energy homeostasis, genes in the catabolic pathways such as *trypsin, zinc proteinase* and *carboxypeptidase* were highly expressed in the Bottom-weight group. While shrimp in the Bottom-weight group lost energy to defense and stress responses, the Top-weight group showed significantly increased levels of genes involved in metabolic and growth such as transcription/translation process (e.g., *asparagine-tRNA ligase*, *collagen alpha-1 (V) chain like* and *acetylcholine receptor subunit alpha-like protein)*. Our observation suggests that shrimp in the Top-weight group were able to allocate their obtained energy mainly toward growth-related pathways, resulting in a better growth performance.

The Top- and the Bottom-weight groups shrimp showed some different metabolite patterns. Significant metabolites found associated with intestine of Bottom-weight shrimp were sugar and plants-derived compounds, suggesting that it could possibly be from plant-based ingredients (such as soybean) in the commercial feed pellets. These metabolites are important nutrient sources for aquaculture (Geurden et al., 2013). However, the excess amount of these compounds in the Bottom-weight shrimp might be due to the limited ability of nutrient absorption by the host shrimp, caused by various factors such as lack of beneficial gut normal flora, biotic and abiotic stresses. Some metabolites found associated with the Top-weight shrimp have been reported to be produced by gut microbes and shrimp itself. For example, short chain fatty acids (e.g., butyric acid) and other lactic acid-derived compounds (3-phenyllactic acid) are synthesized by lactic acid bacteria in the phylum Firmicutes and have been shown to play roles in the health of the host in many aquatic animals (Hoseinifar et al., 2018). In shrimp, effects of butyric acid in diet supplement have been studied to improve the growth performance of *Litopenaeus vannamei* (Silva et al., 2016) and to modulate microbial community in the gut as well as to promote growth performance in gilthead sea bream (*Sparus aurata*) (Rimoldi et al., 2018). *Lactobacillus* as dietary supplement has been reported to enhance immune system in crustaceans such as marron (Foysal et al., 2020), Pacific white shrimp (Zheng et al., 2017) and freshwater prawn (Dash et al., 2014). *Lactobacillus pentosus* has been shown to reduce mortality of *Artemia franciscana* under *Vibrio alginolyticus* challenge (Garcés, Sequeiros & Olivera, 2015). The indole-derived compounds (indole-3-acetic acid and 4-indolecarbaldehyde) and pantothenic acid (vitamin B5) are synthesized by diverse bacteria associated to soil, marine, animal hosts and plants (Hendrikx & Schnabl, 2019; Leonardi & Jackowski, 2007; Patten, Blakney & Coulson, 2013). Indole-3-acetic acid has been reported to have antibiofilm activity against *Vibrio campbellii*, reducing mortality rate in brine and freshwater shrimp cultures (Yang et al., 2017). For pantothenic acid or vitamin B5, it can be obtained from diet and/or can be synthesized by intestinal commensal bacteria (e.g., Bacteroidetes, Firmicutes and Proteobacteria) (Yoshii et al., 2019). It plays a crucial role as a substrate for coenzyme A, an essential co-factor for nutrient absorption and energy production (Lin, Lin & Shiau, 2012). Vitamin B5 was shown to have biological role in maintain growth and physiological function in juvenile blunt snout bream (Qian et al., 2015). Furthermore, some metabolites found associated with the Top-weight group were related to host metabolic pathways. For instance, lysophosphatidylcholine (LPC (18:3)) involves in lipolysis (Chen et al., 2015; Heier et al., 2017), in which LPC, a hydrolyzed form of PC, have been reported to be involved in lipid catabolism in shrimp and crabs from a breakdown of a storage lipid, triacylglycerol by triacylglycerol phospholipase (Kumar et al., 2018). Among available dietary nutrients, lipids yield the highest energy, therefore, efficiency of lipid metabolism would essentially contribute to growth and development in shrimp. Additionally, acylcarnitines eg. palmitoylcarnitine, linoleyl carnitine and oleoyl-L-carnitine, and malic acid were found in significantly higher concentration in the Top-weight shrimp. Acylcarnitines play vital role in balancing sugar and lipid catabolism. They act as carriers for transportation of long-chain fatty acids to catabolize through fatty acid *β*-oxidation, leading to energy production in Zebrafish (Li et al., 2017) and in Chinese mitten crab (Liu et al., 2018). Malic acid is an intermediate metabolite in TCA cycle involved in energy metabolism playing a role on growth performance in crustaceans (Hervant, 1996), fish and crayfish (Skorkowski, 1988). Our observations suggest that the Top-weight shrimp efficiently utilized nutrient sources better than the Bottom-weight shrimp. Furthermore, the multi-omics analyses provide the evidence that both the host and the gut microbiome could synergistically contribute to intestinal metabolites and host metabolism that could impact shrimp growth and development.

## Conclusions

Here, we report the use of multi-omics application to elucidate key microbiota, host pathways and metabolites involving in shrimp growth performance by comparing the results between the Top- and the Bottom-weight shrimp (Fig. 8). The higher growth performance was correlated to the higher abundance of Gram-positive bacteria, mainly Firmicutes. Bacterial network showed higher complexity and competitive interactions in the Bottom-weight shrimp compared to that of the Top-weight shrimp (Fig. 8). Gene expression profiles showed significant upregulation of genes whose encoded products involved in nutrient metabolisms and developmental processes. Interestingly, the immune- and stress-related genes were significantly upregulated in the Bottom-weight shrimp. Moreover, higher concentration of short-chain fatty acids, which was likely to be produced by intestinal bacteria, were found in the intestines of the Top-weight shrimp. Our findings suggested that the elevated expression of immune-related pathways could compromise growth performance, as evidenced by the shrimp in the Bottom-weight group. Further analysis on how metabolites might mediate interaction between microbe and host or vice versa would be necessary to decipher interactions and factors contribute to shrimp growth performance.

##  Supplemental Information

10.7717/peerj.9646/supp-1 Supplemental Information 1 Rarefaction curve analysis for all 16S amplicon sequences obtained from shrimp intestines in Top-weight group (T, *n* = 15) and Bottom-weight group (B, *n* = 15)

10.7717/peerj.9646/supp-2 Supplemental Information 2 Relative abundance of microbial profiles at phylum (A) and genus level (B) in Top-weight group (*n* = 15) and Bottom weight group (*n* = 15) Genera that have relative abundance less than 0.1% were combined and presented as other.

10.7717/peerj.9646/supp-3 Supplemental Information 3 Correlation of gene expression identified with RNA-Seq and RT-qPCR assays Gene expression was determined in log_2 fold-change.

10.7717/peerj.9646/supp-4 Supplemental Information 4 Raw read counts and taxonomy assignment of 16S amplicon sequences obtained from shrimp intestines in Top-weight group (T, *n* = 15) and Bottom-weight group (B, *n* = 15)

10.7717/peerj.9646/supp-5 Supplemental Information 5 Oligonucleotides used in this study for transcriptomic profile validation

10.7717/peerj.9646/supp-6 Supplemental Information 6 Informative features from microbial profiles (53 OTUs), transcriptomic profiles (444 genes) and metabolite profiles (90 metabolites) used for hierarchical clustering analysis

10.7717/peerj.9646/supp-7 Supplemental Information 7 Metabolomes of the Top- (T) and the Bottom- (B) weight groups of 5-month-old *P. monodon* (*n* = 15 per group) Metabolome data were acquired by positive ESI mode (1,004 metabolite features) and by negative ESI mode (360 metabolite features).

10.7717/peerj.9646/supp-8 Supplemental Information 8 Sampling depth analysis of 16S amplicon sequences obtained from shrimp intestines in Top-weight group (T, *n* = 15) and Bottom-weight group (B, *n* = 15) Sequences were clustered into operational taxonomic units (OTUs) at 97% similarity. Two alpha diversity indices including Shannon and Chao1 in each sample are shown. ****

10.7717/peerj.9646/supp-9 Supplemental Information 9 Number of non-redundant transcripts i.e. unigenes that can be annotated using different databases

10.7717/peerj.9646/supp-10 Supplemental Information 10 List of differentially express genes (DEGs) The genes that were enrich by using KOBAS were denote by ‘*’ in front of UnigeneID.****

10.7717/peerj.9646/supp-11 Supplemental Information 11 Metabolite profiles comparing between Top- (T) and Bottom- (B) weight groups of 5-month-old *P. monodon* (*n* = 15/group) The metabolites selected from variable importance in projection (VIP >1) were acquired by positive (58 metabolites) and negative ESI mode (32 metabolites).

## Additional Information and Declarations

### DNA Deposition

The following information was supplied regarding the deposition of DNA sequences: Amplicon sequencing datasets and transcriptome dataset are available via NCBI SRA database under accession numbers: PRJNA553862.

### Data Availability

The following information was supplied regarding data availability: Amplicon sequencing datasets and transcriptome dataset are available at NCBI SRA: PRJNA553862. Metabolite metadata are available in Table S4.
https://www.ncbi.nlm.nih.gov/bioproject/PRJNA553862.
