# Supplementary figures and images for: Multi-omics analysis to examine microbiota, host gene expression and metabolites in the intestine of black tiger shrimp (Penaeus monodon) with different growth performance
Source: PeerJ. 2020 Aug 14;8:e9646. doi: 10.7717/peerj.9646 (PMC7430268; doi:10.7717/peerj.9646)

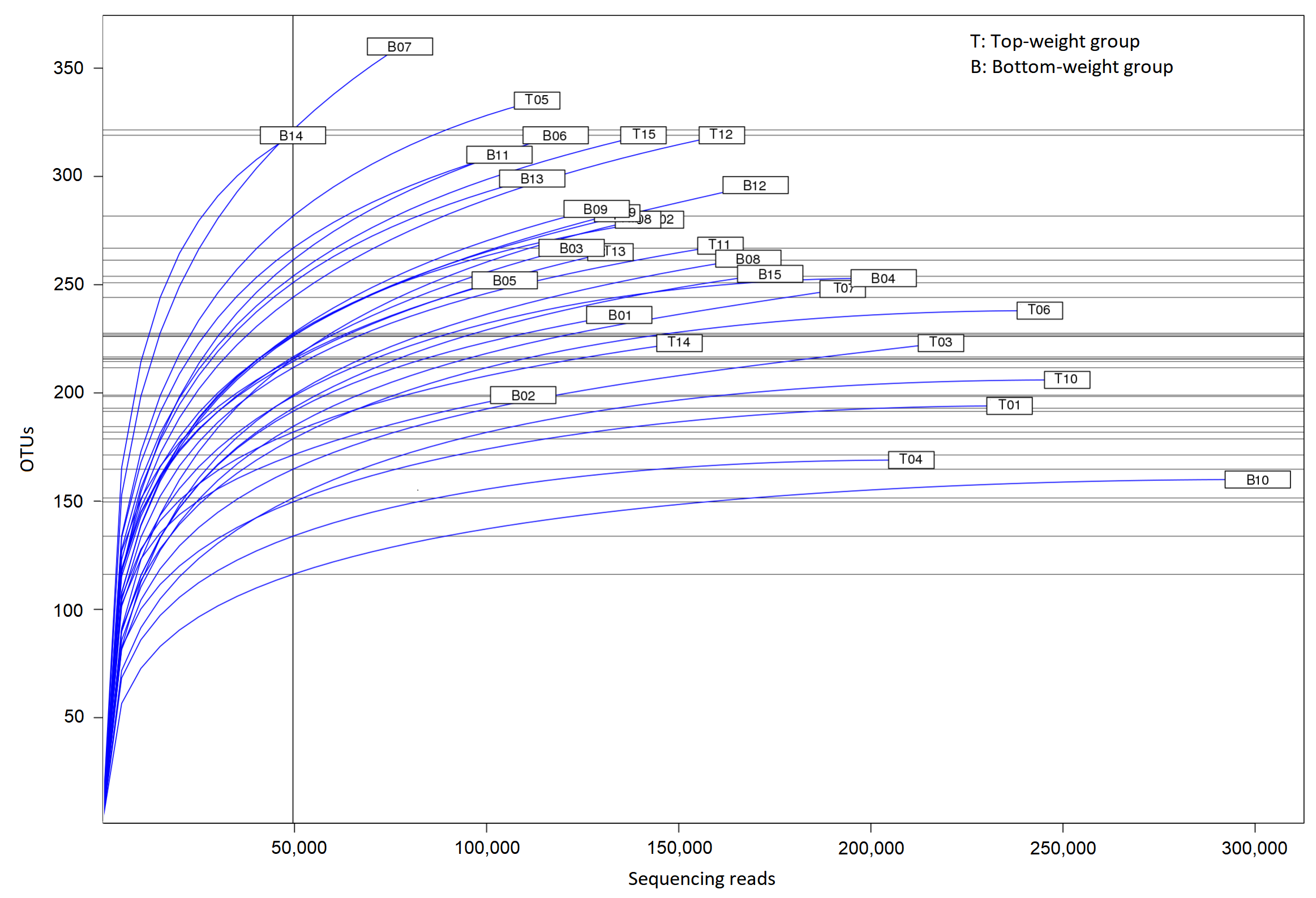

Supplement: Supplemental Information 1 [file peerj-08-9646-s001.png]

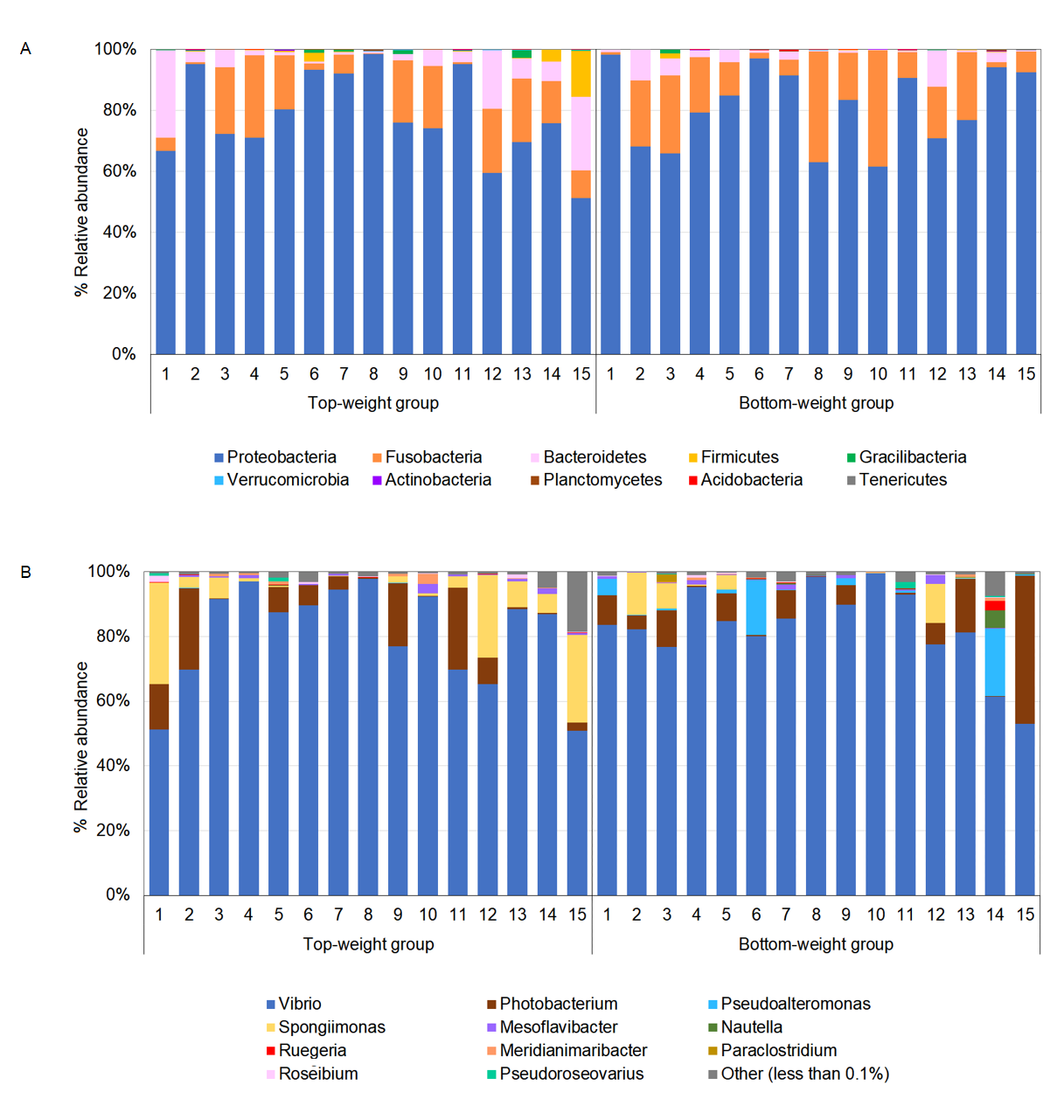

Supplement: Supplemental Information 2 — Genera that have relative abundance less than 0.1% were combined and presented as other. [file peerj-08-9646-s002.png]

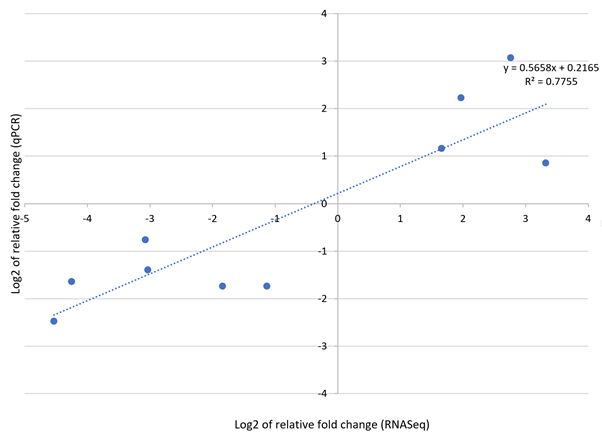

Supplement: Supplemental Information 3 — Gene expression was determined in log_2 fold-change. [file peerj-08-9646-s003.png]
