# Supplementary material for: Multi-omics analysis to examine microbiota, host gene expression and metabolites in the intestine of black tiger shrimp (Penaeus monodon) with different growth performance
Source: PeerJ. 2020 Aug 14;8:e9646. doi: 10.7717/peerj.9646 (PMC7430268; doi:10.7717/peerj.9646)
Supplement: Supplemental Information 5 [file peerj-08-9646-s005.docx]

**Table S2**. Oligonucleotides used in this study for transcriptomic profile validation.

| Gene name | Primer | Sequence 5’-3’ | Amplicon size (bp) |
| --- | --- | --- | --- |
| Serine protease | SP-F  SP-R | CGTCTTGAAGGAGGTTGAGC  GTGAAGGATGGGTGCAGTTT | 105 |
| Heat shock protein 40 | Hsp40-F  Hsp40-R | ACGTTTTCGAGGACATGGAC  GACGACCCTTGCTCTCGTAG | 144 |
| Alpha-aminoadipic semialdehyde synthase | AASS-F  AASS-R | TGCATATTGGACCTGCACAT  CTTGGGACACGTTTCCAGTT | 150 |
| Unknown | Unk-F  Unk-R | TACGCATCGGGTGACAAGTG  TGATCTTTCGCATGACCGCA | 83 |
| Hemocyte homeostasis-associated protein | HHAP-F  HHAP-R | TACCAGGGCAACAGGAGAAC  GTGTTGCACGATTTGCACTT | 113 |
| Transglutaminase | TGM-F  TGM-R | GACAGTGCCACTCGAAAACA  TGCACAGCTCCCTACTGTTG | 119 |
| C-type lectin | Lec-F  Lec-R | AGTGCTGGACGAGTGCTTCT  GCATAGACGTTCCTGGGTGT | 111 |
| Reverse transcriptase | RT-F  RT-R | TGACTGTGGTTGTTGCTGGT  TTCATGAAGAAGCCCCACTC | 129 |
| Chitinase 1 | Chi1-F  Chi1-R | TGTTGCTCCCACTCTTCCTC  TAGGCCCCATAGTTGTGGTC | 134 |
| Cytochrome c oxidase III | COX3-F  COX3-R | CCGAAAATCCTTTCCAGAGACCT  GCATGGTGAGCTAAAGTGACAG | 82 |
